# Obsessive-compulsive disorder in the World Mental Health surveys

**DOI:** 10.1186/s12916-025-04209-5

**Published:** 2025-07-09

**Authors:** Dan J. Stein, Ayelet Meron Ruscio, Yasmin Altwaijri, Wai Tat Chiu, Nancy A. Sampson, Sergio Aguilar-Gaxiola, Ali Al-Hamzawi, Jordi Alonso, Stephanie Chardoul, Oye Gureje, Chiyi Hu, Elie G. Karam, John J. McGrath, Fernando Navarro-Mateu, Kate M. Scott, Juan Carlos Stagnaro, Yolanda Torres, Cristian Vladescu, Jacek Wciórka, Miguel Xavier, Ronald C. Kessler, the World Mental Health Survey collaborators, Dan J. Stein, Sergio Aguilar-Gaxiola, Ali Al-Hamzawi, Jordi Alonso, Stephanie Chardoul, Oye Gureje, Elie G. Karam, John J. McGrath, Fernando Navarro-Mateu, Kate M. Scott, Juan Carlos Stagnaro, Cristian Vladescu, Miguel Xavier, Ronald C. Kessler, Yasmin A. Altwaijri, Laura Helena Andrade, Lukoye Atwoli, Corina Benjet, Guilherme Borges, Ronny Bruffaerts, Brendan Bunting, Jose Miguel Caldas-de-Almeida, Graça Cardoso, Louisa Degenhardt, Giovanni de Girolamo, Josep Maria Haro, Meredith G. Harris, Hristo Hinkov, Chiyi Hu, Peter de Jonge, Aimee Nasser Karam, Georges Karam, Alan E. Kazdin, Norito Kawakami, Andrzej Kiejna, Viviane Kovess-Masfety, Maria Elena Medina-Mora, Jacek Moskalewicz, Daisuke Nishi, Marina Piazza, José Posada-Villa, Annelieke Roest, Margreet ten Have, Nathan Tintle, Maria Carmen Viana, Daniel V. Vigo, David R. Williams, Bogdan Wojtyniak, Alan M. Zaslavsky

**Affiliations:** 1 SAMRC Unit On Risk & Resilience in Mental Disorders, Dept of Psychiatry & Neuroscience Institute, University of Cape Town, Cape Town, South Africa; 2 Department of Psychology, University of Pennsylvania, Philadelphia, PA USA; 3 Biostatistics, Epidemiology and Scientific Computing Department, King Faisal Specialist Hospital & Research Center, Riyadh, Saudi Arabia; 4 Department of Health Care Policy, Harvard Medical School, Boston, MA USA; 5 Department of Internal Medicine, Center for Reducing Health Disparities, UC Davis Health System, Sacramento, CA USA; 6 College of Medicine, University of Al-Qadisiya, Al Diwaniyah, Diwaniya Governorate Iraq; 7 Health Services Research Unit, Hospital del Mar Medical Research Institute (IMIM), Barcelona, Spain; 8 Department of Medicine and Life Sciences, Pompeu Fabra University (UPF), Barcelona, Spain; 9 Centro de Investigación Biomédica en Red de Epidemiología y Salud Pública (CIBERESP), Madrid, Spain; 10 Survey Research Center, Institute for Social Research, University of Michigan, Ann Arbor, MI USA; 11 Department of Psychiatry, University College Hospital, Ibadan, Nigeria; 12 Department of Psychiatry, Stellenbosch University, Cape Town, South Africa; 13 Shenzhen Institute of Mental Health & Shenzhen Kangning Hospital, Shenzhen, China; 14 Institute for Development, Research, Advocacy and Applied Care (IDRAAC), ResearchBeirut, Lebanon; 15 Department of Psychiatry and Clinical Psychology, St George Hospital University Medical Center, Beirut, Lebanon; 16 Queensland Centre for Mental Health Research, The Park Centre for Mental Health, Wacol, QLD Australia; 17 Queensland Brain Institute, The University of Queensland, St Lucia, QLD Australia; 18 National Centre for Register-Based Research, Aarhus University, Aarhus, Denmark; 19 Unidad de Docencia, Investigacion y Formación en Salud Mental (UDIF-SM), Servicio Murciano de Salud, Murcia Health Service, C/Lorca, nº 58. - El Palmar, Murcia, 30120 Spain; 20 Instituto Murciano de Investigación Biosanitaria - Pascual Parrilla (IMIB-Pascual Parrilla), Hospital Virgen de la Arrixaca, El Palmar, Murcia, 30120 Spain; 21 Centro de Investigación Biomédica en ERed en Epidemíologia y Salud Pública (CIBERESP), Madrid, 28029 Spain; 22 Department of Psychological Medicine, University of Otago, Dunedin, Otago New Zealand; 23 Departamento de Psiquiatría y Salud Mental, Facultad de Medicina, Universidad de Buenos Aires, Buenos Aires, Argentina; 24 Center for Excellence On Research in Mental Health, CES University, Medellín, Colombia; 25 National Institute for Health Services Management, Bucharest, Romania; 26 University Titu Maiorescu, Bucharest, Romania; 27 Department of Psychiatry, Institute of Psychiatry and Neurology, Warsaw, Poland; 28 Nova Medical School, Nova University of Lisbon, Lisbon, Portugal

**Keywords:** Obsessive-compulsive disorder, Community epidemiology, Global mental health, World Mental Health surveys

## Abstract

**Background:**

National surveys have suggested that obsessive-compulsive disorder (OCD) is a prevalent and impairing condition. However, there are few cross-national data on OCD, with data particularly scarce in low- and middle-income countries. Here we employ data from the World Mental Health surveys to characterize the onset, course, severity, and treatment of OCD across a range of countries in different geographic regions of the world.

**Methods:**

Data came from general population surveys carried out in 10 countries (Argentina, Australia, Colombia, Iraq, Poland, People’s Republic of China, Portugal, Romania, Saudi Arabia, Spain) using a consistent research protocol and interview. A total of 26,136 adults were assessed for OCD in face-to-face interviews and were included in the present analyses. We examined lifetime and 12-month prevalence as well as age of onset, persistence, severity, and treatment of *DSM-IV* OCD in six high-income countries (HICs) and four low- or middle-income countries (LMICs). We also investigated socio-demographic variables and temporally prior mental disorders as predictors of OCD onset, persistence, severity, and treatment.

**Results:**

Across the 10 countries surveyed, OCD has a combined lifetime prevalence of 4.1%. The 12-month prevalence (3.0%) is nearly as high, suggesting a highly persistent course of illness. Age of onset is early, with more than 80% of OCD cases beginning by early adulthood. Most OCD cases in the community are mild (47.0%) or very mild (27.5%), with a smaller percentage designated as moderate (22.9%) or severe (2.7%) by the Yale-Brown Obsessive-Compulsive Scale. Only 19.8% of respondents with OCD received any mental health treatment in the past year, with treatment rates much higher in HICs (40.5%) than LMICs (7.0%). Cross-nationally, OCD commonly emerges in adolescence or early adulthood against a backdrop of earlier-occurring mental disorders. With few exceptions (e.g., prior social phobia), the socio-demographic and psychopathological risk factors for OCD onset, persistence, severity, and treatment are distinct.

**Conclusions:**

These cross-national data underscore clinical lessons regarding the importance of early diagnosis of OCD and comprehensive evaluation of comorbidity; draw attention to OCD as an undertreated disorder, particularly in LMIC contexts; and emphasize the public health significance of this often-overlooked condition.

**Supplementary Information:**

The online version contains supplementary material available at 10.1186/s12916-025-04209-5.

## Background

Obsessive-compulsive disorder (OCD) was traditionally considered a relatively rare condition in the general population and in mental health clinics [1–3]. Advances in psychiatric epidemiology, including the use of structured interviews based on *Diagnostic and Statistical Manual of Mental Disorders* (*DSM*) diagnostic criteria, overturned this view. In the 1980s, the Epidemiological Catchment Area (ECA) survey in the USA used the Diagnostic Interview Schedule (DIS) to assess *DSM-III* OCD and found a lifetime prevalence of 2.3% [3]. Surveys employing this instrument in six other high-income countries indicated that most lifetime prevalence rates fell within the range of 1.9% (in Korea) to 2.5% (in Puerto Rico) [4].

The World Health Organization Composite International Diagnostic Interview (CIDI) incorporated additional advances, including the use of dimensional symptom severity measures [5]. Employing the CIDI, the National Comorbidity Survey Replication (NCS-R) in the USA found a lifetime prevalence of 2.3% for *DSM-IV* OCD, while also observing a high prevalence of subthreshold OCD symptoms in the population [6]. Additional key findings were that OCD typically emerges against a backdrop of temporally prior mental disorders and that the severity of OCD is robustly associated with role impairment, with severe OCD cases reporting more severe disability in occupational and social domains, and substantially more days out of role, than moderate cases [6].

Despite these advances, several aspects of the epidemiology of OCD require further attention [1, 7]. First, there are very few representative surveys in non-Western countries—especially in low- and middle-income countries, where the large majority of the world’s population resides [1, 8]. Surveys of this sort are needed given well-documented limitations of generalizing behavioral research findings from Western, Educated, Industrialized, Rich, and Democratic (WEIRD) societies to other parts of the world [9]. Second, with the exception of early work using the DIS, there has been a dearth of cross-national research on the epidemiology of OCD. In fact, no cross-national study has used the improved *DSM-IV* diagnostic criteria [10], which were largely unchanged in *DSM-5* [11]. Investigating OCD across cultures provides an opportunity to identify more universal features and correlates of the disorder than can be observed when studying individual cultures [12]. It also capitalizes on the larger sample sizes—and corresponding statistical power—afforded by aggregating OCD cases across surveys. Third, while there has been some research on correlates of OCD onset in the community [7], little epidemiological research has considered correlates of other important outcomes, such as OCD persistence, severity, and treatment. Moreover, with the exception of the NCS-R [6], past research has focused on cross-sectional associations between correlates and OCD. This has made it difficult to determine which correlates precede the onset of OCD and consequently reflect risk factors for OCD-related outcomes [13].

The World Mental Health (WMH) surveys, the world’s largest set of coordinated community surveys of mental disorders [14], allow detailed investigation of the epidemiology of OCD. Here we employed data from the WMH surveys to characterize the onset, course, severity, and treatment of OCD across a range of high-income and low- and middle-income countries in different geographic regions of the world. Then, using an aggregated cross-national dataset, we evaluated temporally prior variables, including socio-demographic characteristics and earlier mental disorders, as predictors of subsequent OCD-related outcomes.

## Methods

### Sample

Data came from single-interview WMH surveys administered between 2005 and 2019 in 10 countries (Table 1). Six of the surveys were administered in countries classified by the World Bank as high-income (HICs) at the time of data collection; they included nationally representative surveys in Argentina, Australia, Poland, Portugal, and Saudi Arabia, as well as a regional survey in Murcia, Spain. Four of the surveys were administered in countries classified by the World Bank as low- or middle-income (LMICs); they included nationally representative surveys in Iraq and Romania, as well as regional surveys in Medellin, Colombia and Shenzhen, People’s Republic of China. The combined sample size across surveys was *N* = 26,136. All respondents were age 18 or older at the time of the survey.

**Table 1:** Prevalence of *DSM-IV* OCD in the World Mental Health surveys

Country	Lifetime prevalence		12-month prevalence		12-month prevalence among lifetime cases		
	%	(SE)	%	(SE)	%	(SE)	(*n*)
I. Low- or middle-income countries (LMIC)	4.9	(0.3)	3.9	(0.3)	80.3	(2.1)	(12,567)
Medellin, Colombia	2.8	(0.8)	2.4	(0.8)	85.3	(6.7)	(541)
Iraq	4.6	(0.5)	3.6	(0.4)	79.7	(5.1)	(4,332)
Shenzhen, People’s Republic of China	5.5	(0.4)	4.5	(0.4)	80.4	(2.1)	(7,132)
Romania	0.6	(0.3)	0.5	(0.3)	82.0	(12.5)	(562)
II. High-income countries (HIC)	3.4	(0.2)	2.2	(0.1)	65.9	(3.3)	(13,569)
Argentina	2.9	(0.8)	2.5	(0.8)	86.3	(7.5)	(692)
Australia	3.7	(0.3)	2.6	(0.2)	70.7	(4.2)	(8,463)
Poland	1.3	(0.4)	0.3	(0.1)	23.1	(9.6)	(1,607)
Portugal	5.3	(1.1)	4.4	(0.9)	82.4	(7.7)	(539)
Saudi Arabia	4.0	(0.7)	1.8	(0.4)	45.1	(7.2)	(1,789)
Murcia, Spain	0.4	(0.3)	0.3	(0.2)	70.7	(17.1)	(479)
III. Total sample	4.1	(0.2)	3.0	(0.1)	74.1	(1.9)	(26,136)

*Abbreviations* *OCD* obsessive-compulsive disorder, *SE* standard error

All surveys used multistage clustered area probability household sample designs. Response rates for individual surveys ranged from 60.0 to 97.2%, with a weighted (by sample size) average response rate across surveys of 70.9% (Additional File 1 Appendix Table 1). Interviews were conducted face-to-face in respondents’ homes. Other than in Australia, Iraq, and Romania, the interview was divided into two parts. Part 1 assessed core disorders (as detailed below in the section on Comorbid Disorders) and was administered to all respondents. Part 2 addressed additional disorders, including OCD, and correlates, and was administered to all Part 1 respondents who met criteria for any lifetime Part 1 disorder plus a probability subsample of other respondents. The reason for this division is that, as most respondents do not have a history of mental disorder, interviewing all non-cases in full is unnecessary to maximize statistical power for case-control comparisons. Significant efficiencies can therefore be achieved by ending the interview early for a random subsample of non-cases once it is determined that they do not meet criteria for any mental disorder [15]. Part 1 data were weighted to adjust for differential probabilities of selection and to match population distributions on Census socio-demographic and geographic distributions. Part 2 data were additionally weighted for the undersampling of Part 1 respondents without core disorders.

### Measures

#### Interview

Trained lay interviewers administered a fully structured diagnostic interview, the World Health Organization Composite International Diagnostic Interview Version 3.0 (CIDI 3.0) [5]. The interview and training materials were developed in English and subsequently translated into other languages using a comprehensive protocol designed to achieve both conceptual equivalence and measurement consistency across countries. High-quality translations were obtained through a structured six-step process: (1) forward translation; (2) expert panel review; (3) independent back-translation; (4) harmonization of terminology and phrasing across countries sharing the same language; (5) pretesting with cognitive interviewing; and (6) final revision, production, and documentation of the translated instrument [16]. Interviewers were required to complete a standardized training course successfully before they could undertake fieldwork and collect data for the study. Consistent procedures were then used across surveys to check interviewer accuracy and implement standardized data cleaning and coding procedures [17]. Informed consent was obtained before starting the interview. Local institutional review committees approved and monitored the surveys to ensure protection of human subjects per international and local guidelines. All procedures contributing to this work complied with the ethical standards of the relevant national and institutional committees on human experimentation.

#### OCD diagnosis, onset, persistence, and severity

OCD was assessed using an updated version of CIDI 3.0 that improved the skip logic of the OCD section to conform more closely to *DSM-IV* diagnostic criteria than earlier CIDI versions. All countries that administered this improved OCD section were included in the present analyses. The OCD section assessed lifetime experiences of contamination, harming, ordering, hoarding, and “other” obsessions and compulsions (O/C). Respondents endorsing one or more of the O/C types were asked about time spent on obsessions (described as *repeated unpleasant thoughts*,* images*,* or impulses*) and compulsions (described as *behaviors people feel driven to do over and over, either physically or in their mind*). Subsequent questions assessed any reported obsessions for all other diagnostic criteria of OCD, followed by a parallel series of questions for any reported compulsions. *DSM-IV* general medical and substance-related exclusions, but not diagnostic hierarchy rules, were applied in making diagnoses. Respondents who met *DSM-IV* criteria for OCD in their lifetime were asked about their age when they first had significant obsessions or compulsions that either took up at least 1 h per day, caused distress, or interfered with their day-to-day activities, as well as their age when they most recently experienced obsessions or compulsions most days for at least 2 weeks. Persistence was defined as the proportion of respondents with lifetime OCD who qualified for the disorder in the past 12 months. A clinical reappraisal study carried out with a subset of NCS-R respondents [6] showed that CIDI OCD diagnoses had excellent individual-level concordance with diagnoses assigned by clinical interviewers using the Structured Clinical Interview for *DSM-IV* (SCID) [18], with an area under the receiver operating characteristic curve of 0.95 and a kappa of 0.90 (*SE* = 0.03). Sensitivity was 90.2, specificity was 99.7, and total classification accuracy was 99.5.

Clinical severity of 12-month OCD cases was assessed using a fully structured version of the Yale-Brown Obsessive-Compulsive Scale (Y-BOCS) (question wording can be found at [19]). The Y-BOCS is the clinical standard for assessing the severity of OCD [20]. As several respondents reported either obsessions without compulsions or compulsions without obsessions and consequently were administered only half of the Y-BOCS, we calculated severity scores for all respondents by taking the higher of the Y-BOCS obsessions and compulsions subscale scores, then doubling this score to arrive at the standard Y-BOCS metric familiar to researchers and clinicians. This approach, previously employed in the NCS-R [6], is supported by work showing that with good clinical probing obsessions and compulsions almost always co-exist, and that severity scores on the Y-BOCS obsessions and compulsions subscales tend to be similar [10]. Different severity cut points have been proposed for the Y-BOCS. To capture OCD cases across the full severity continuum, we adopted an approach that includes very mild scores (0–7) as well as scores for mild (8–15), moderate (16–24), and severe (24–31) symptoms [21].

#### Comorbid disorders

CIDI 3.0 assesses lifetime and 12-month mental disorders using *DSM-IV* diagnostic criteria. Blinded clinical reappraisal studies carried out in Asia [22, 23], Europe [24], Latin America [25], the Middle East [26], and the USA [27] have found consistently good concordance between diagnoses based on the CIDI and diagnoses based on blinded gold-standard clinical interviews using the SCID.

We analyzed all disorders that were assessed by at least nine of the 10 countries in the current report. Aside from OCD, this included four anxiety disorders (panic disorder with or without agoraphobia, social phobia, generalized anxiety disorder, posttraumatic stress disorder), two mood disorders (major depressive disorder and bipolar spectrum disorder, the latter of which included bipolar I disorder, bipolar II disorder, and subthreshold bipolar disorder), two substance use disorders (alcohol and drug use disorders, each of which included abuse and dependence), and one externalizing disorder (attention-deficit/hyperactivity disorder; ADHD). Respondents meeting lifetime criteria for any disorder were asked about age of onset using a question series designed to facilitate accurate dating [28]; responses were used to determine the temporal order of each disorder vis-à-vis OCD.

#### Socio-demographic characteristics

Several socio-demographic variables were examined as predictors of OCD. They included the respondent’s *sex* (male/female) and *age at interview* (divided into categories of 18–29 years, 30–44 years, 45–59 years, and 60+ years). They also included other variables defined in relation to the age of onset of OCD. *Age of onset* was a continuous variable representing the respondent’s age when OCD began, whereas *time since onset* represented the number of years between age of onset and age at interview. *Education* was categorized by years of matriculation completed before OCD onset: low (0–6 years), low‐average (7–9 years), high‐average (10–15 years), and high (16+ years), with a separate category included for respondents who were enrolled as a student when OCD began. *Marital status* at OCD onset included categories of never married, married, or previously married, with the latter category including respondents who were separated, divorced, or widowed.

#### Treatment

Twelve-month treatment estimates were obtained by asking respondents if they received treatment for any mental or behavioral problem in the past year. Summary measures of 12-month treatment were created separately for services received in the *health care* sector (further subdivided into *general medical* and *mental health specialty*) and the *non-health care* sector (further subdivided into *human services* and *complementary-alternative medicine*), as well as for services received in *any* sector in the year before the interview.

### Statistical analysis

Weights were applied to the data to adjust for differences in within-household probabilities of selection and to calibrate the samples to match Census population distributions on socio-demographic and geographic variables. Part 2 data were also weighted to adjust for differential probabilities of selection into Part 2. The Taylor series linearization method, implemented in SAS 9.4 [29], was used to adjust standard errors for the effects of these weights as well as for the effects of geographic clustering of the WMH data.

Cross-tabulations were used to estimate the prevalence, persistence, and severity of OCD, and to examine treatment seeking as a function of severity. Age-of-onset distributions were estimated separately for males and females with OCD using the actuarial method. Discrete-time survival analysis with person-year as the unit of analysis [30] was used to predict lifetime OCD. Each survival model tested whether the respondent’s status on a given socio-demographic variable or comorbid disorder in the year before OCD onset was associated with the first onset of OCD the following year, controlling for all other variables in the model. By contrast, person-level logistic regression analysis was used to predict 12-month OCD outcomes, including persistence, severity, and treatment. These person-level models examined whether the respondent’s status on a given socio-demographic variable or comorbid disorder at the time that OCD began was associated with OCD-related outcomes in the year before interview. The temporal order of OCD and other variables was determined from retrospective age-of-onset reports. Survival coefficients were transformed to odds ratios (ORs) with 95% confidence intervals (CIs) for ease of interpretation. Statistical significance was evaluated at the 0.05 level using two-sided tests.

## Results

### Prevalence

Across all surveys, *DSM-IV* OCD has a combined lifetime prevalence of 4.1% and a 12-month prevalence of 3.0% (Table 1). Lifetime prevalence varies widely across countries, ranging from a low of 0.4% in Murcia, Spain to a high of 5.5% in Shenzhen, People’s Republic of China. Although there is also wide variability within country groups, the overall pattern is for lifetime prevalence to be higher in LMICs (4.9%) compared to HICs (3.4%; *χ*^2^_1_ = 16.9, *p* < 0.001). The pattern is very similar for 12-month prevalence, which ranges from 0.3 to 4.5% across countries yet is higher, on average, in LMICs (3.9%) than HICs (2.2%; *χ*^2^_1_ = 27.3, *p* < 0.001).

These estimates, which focus on diagnosed cases, miss the far larger number of individuals with subclinical obsessions and compulsions in the population (Additional File 1 Appendix Table 2). Fully 13.6% of the cross-national sample reported lifetime experiences of O/C. Most respondents reported only one type of O/C, most commonly involving harming (6.5%) or hoarding (5.9%). Type of O/C is only weakly associated with conditional probability of lifetime OCD, which ranges from a low of 35.5% (for hoarding) to a high of 48.3% (for “other” O/C whose content was not specified by respondents). By contrast, number of O/C is strongly associated with conditional probability of OCD, which increases monotonically from 16.9% of respondents who reported a single type to 67.4% of respondents who reported all five types assessed here. Among individuals with OCD, those reporting different O/C types do not differ markedly in persistence (72.2–76.6% of lifetime cases have 12-month OCD) or severity (28.9–38.5% of 12-month cases are moderate to severe). Instead, severity increases with number of O/C types, with moderate to severe OCD found in 10.9% of 12-month cases involving one type compared to 50.6% of cases involving all five types.

### Course

**Fig. 1:**
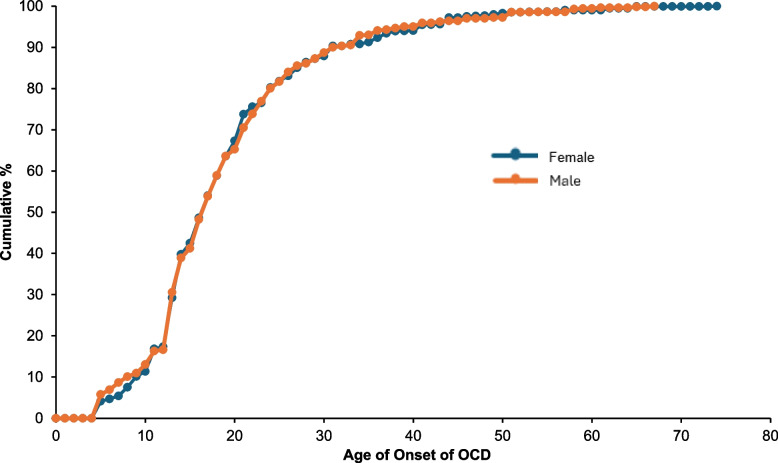
Age of onset of OCD by sex Abbreviation OCD obsessive-compulsive disorder Cumulative age-of-onset distributions for first obsession or compulsion, shown separately for female and male respondents with lifetime OCD

OCD typically emerges early in life (Fig. 1). First onsets most often occur in adolescence or early adulthood, with half of cases beginning by age 17 and more than 80% beginning by age 24. There is no evidence of sex differences in age of onset, with males and females having very similar age-of-onset curves.

Once OCD begins, it is highly persistent (Table 1). Nearly three-quarters (74.1%) of respondents with lifetime OCD still qualified for the disorder in the 12 months before the interview. Although persistence is lower on average in HICs (65.9%) than in LMICs (80.3%), this difference is driven mainly by two countries with very low persistence rates, Poland (23.1%) and Saudi Arabia (45.1%). In the eight other countries, persistence is the norm, reported by 70.7–86.3% of lifetime cases.

### Severity

In this community sample, most 12-month OCD cases score in the mild (47.0%) or very mild (27.5%) range on the Y-BOCS (Table 2). Another 22.9% of cases score in the moderate range, with fewer than 3% scoring as severe. OCD severity varies across countries: The modal 12-month case is very mild in Medellin, Colombia; mild in Australia; and moderate in Saudi Arabia. On average, OCD is more severe in HICs than in LMICs. This difference is driven mainly by very mild cases, which are more common in LMICs (32.4%) than in HICs (19.7%; *χ*^2^_1_ = 8.2, *p* = 0.004).

**Table 2:** Severity of 12-month *DSM-IV* OCD by country and country income group^a^

Country	Severe		Moderate		Mild		Very Mild		
	%	(SE)	%	(SE)	%	(SE)	%	(SE)	(n)
I. Low- or middle-income countries (LMIC)	1.1	(0.8)	20.7	(2.7)	45.8	(3.0)	32.4	(3.4)	(420)
Medellin, Colombia	0.0	(0.0)	19.9	(9.0)	32.9	(12.6)	47.2	(13.7)	(19)
Iraq	3.4	(2.5)	23.0	(4.7)	38.3	(5.8)	35.2	(6.8)	(132)
Shenzhen, People’s Republic of China	0.0	(0.0)	19.7	(3.4)	50.2	(3.5)	30.1	(4.0)	(265)
Romania	0.0	(0.0)	15.1	(15.3)	23.6	(21.9)	61.3	(26.0)	(4)
II. High-income countries (HIC)	5.2	(2.2)	26.3	(3.0)	48.9	(3.8)	19.7	(2.7)	(355)
Argentina	0.0	(0.0)	18.7	(7.2)	53.9	(11.8)	27.4	(10.7)	(27)
Australia	6.1	(3.0)	21.0	(3.3)	51.7	(4.7)	21.2	(3.4)	(218)
Poland	0.0	(0.0)	7.0	(7.2)	68.1	(10.3)	24.8	(8.4)	(12)
Portugal	2.0	(2.0)	23.9	(9.9)	52.1	(10.8)	22.0	(8.6)	(35)
Saudi Arabia	4.9	(3.3)	72.8	(9.5)	22.3	(9.3)	0.0	(0.0)	(60)
Murcia, Spain	0.0	(0.0)	0.0	(0.0)	24.5	(24.0)	75.5	(24.0)	(3)
III. Total sample	2.7	(1.0)	22.9	(2.0)	47.0	(2.4)	27.5	(2.3)	(775)

*Abbreviations* *OCD* obsessive-compulsive disorder, *SE* standard error

^a^The severity categories are based on a scheme developed by [21], which divides scores on the Yale-Brown Obsessive-Compulsive Scale (Y-BOCS) into five categories: Extreme (Y-BOCS = 32–40), Severe (Y-BOCS = 24–31), Moderate (Y-BOCS = 16–23), Mild (Y-BOCS = 8–15), and subthreshold (Y-BOCS = 0–7). The subthreshold category is re-labeled Very Mild here because all 12-month OCD cases met the *DSM-IV* diagnostic threshold for the disorder. The Extreme category is omitted because none of the World Mental Health survey respondents had a Y-BOCS score > 32

### Treatment

Cross-nationally, only 19.8% of respondents with 12-month OCD received treatment in the past year (Table 3). Individuals with more severe OCD are more likely to receive treatment: Treatment rates are highest among severe cases (52.5%), lower among moderate (20.9%) and mild (22.7%) cases, and lowest among very mild cases (10.7%; *χ*^2^_3_ = 10.4, *p* < 0.016). This general pattern is evident within each treatment sector but reaches statistical significance only in the specialty mental health, complementary-alternative medicine, and any non-health care sectors (*χ*^2^_3_ = 8.4–14.1, *p* = 0.003–0.041).

**Table 3:** Treatment seeking associated with 12-month *DSM-IV* OCD as a function of OCD severity and country income group^a^

Treatment sector	Severe		Moderate		Mild		Very Mild		All 12-month OCD cases		
	%	(SE)	%	(SE)	%	(SE)	%	(SE)	%	(SE)	*χ﻿*^2^_3_
I. Health care											
a. Specialty mental health^b^											
LMIC	1.9	(2.4)	2.9	(1.3)	1.8	(1.5)	1.7	(1.2)	2.0	(0.8)	0.5
HIC	53.4	(21.9)	20.3*	(4.9)	35.1*	(4.8)	15.0*	(6.1)	28.2*	(3.4)	9.0**
Total	40.2	(20.4)	10.5	(2.4)	15.0	(2.6)	5.3	(2.0)	12.0	(1.6)	10.4**
b. General medical^c^											
LMIC	9.7	(10.4)	2.3	(1.2)	1.7	(1.5)	3.3	(2.6)	2.4	(1.1)	1.2
HIC	58.1	(18.5)	23.4*	(4.8)	24.1*	(3.6)	15.3*	(4.7)	23.9*	(3.0)	3.5
Total	45.7	(18.9)	11.5	(2.3)	10.6	(1.9)	6.6	(2.3)	10.6	(1.4)	4.6
c. Any health care^d^											
LMIC	9.7	(10.4)	5.1	(2.1)	2.1	(1.5)	4.5	(2.8)	3.6	(1.2)	2.1
HIC	64.5	(16.7)	33.2*	(6.0)	44.4*	(5.1)	26.6*	(7.3)	39.0*	(3.5)	5.5
Total	50.5	(18.4)	17.4	(3.1)	18.9	(2.9)	10.5	(2.9)	17.1	(1.8)	6.5
II. Non-health care											
a. Human services^e^											
LMIC	7.8	(9.4)	1.1	(0.7)	2.5	(1.9)	0.0	(0.0)	1.5	(0.9)	-
HIC	38.7	(25.5)	1.8	(1.2)	5.0	(1.7)	0.0	(0.0)	4.9	(2.2)	-
Total	30.8	(21.7)	1.4	(0.6)	3.5	(1.3)	0.0	(0.0)	2.8	(1.0)	-
b. CAM^f^											
LMIC	0.0	(0.0)	4.6	(3.3)	3.0	(1.7)	0.2	(0.2)	2.4	(1.0)	-
HIC	38.7	(25.5)	6.7	(2.3)	6.4	(2.6)	2.7	(2.0)	7.4	(2.5)	2.6
Total	28.8	(22.0)	5.5	(2.1)	4.3	(1.5)	0.9	(0.6)	4.3	(1.1)	8.4**
c. Any non-health care^g^											
LMIC	7.8	(9.4)	5.7	(3.3)	5.1	(2.5)	0.2	(0.2)	3.7	(1.3)	7.0
HIC	38.7	(25.5)	8.5	(2.6)	10.0	(2.9)	2.7	(2.0)	9.7*	(2.5)	4.8
Total	30.8	(21.7)	6.9	(2.2)	7.1	(1.9)	0.9	(0.6)	6.0	(1.3)	14.1**
III. Any treatment^h^											
LMIC	17.5	(16.3)	9.2	(3.8)	7.3	(3.0)	4.7	(2.8)	7.0	(1.8)	1.8
HIC	64.5	(16.7)	35.8*	(6.1)	46.1*	(5.0)	26.6*	(7.3)	40.5*	(3.5)	5.9
Total	52.5	(18.2)	20.9	(3.6)	22.7	(3.2)	10.7	(2.9)	19.8	(1.9)	10.4**

*Abbreviations* *OCD* obsessive-compulsive disorder, *SE* standard error, *LMIC* low- or middle-income countries, *HIC* high-income countries, *CAM* complementary-alternative medicine

^a^The severity categories are based on a scheme developed by [21], which divides scores on the Yale-Brown Obsessive-Compulsive Scale (Y-BOCS) into five categories: Extreme (Y-BOCS = 32–40), Severe (Y-BOCS = 24–31), Moderate (Y-BOCS = 16–23), Mild (Y-BOCS = 8–15), and subthreshold (Y-BOCS = 0–7). The subthreshold category is re-labeled Very Mild here because all 12-month OCD cases met the *DSM-IV* diagnostic threshold for the disorder. The Extreme category is omitted because none of the World Mental Health survey respondents had a Y-BOCS score > 32

^b^Includes psychiatrist, psychologist, or other mental health professional; social worker or counselor in a mental health specialty setting; use of a mental health helpline; or overnight admission for a mental health, drug, or alcohol problem, with a presumption of daily contact with a psychiatrist

^c^Includes general practitioner, other medical doctor, nurse, occupational therapist, or other health care professional not previously mentioned

^d^Includes the specialty mental health sector or the general medical sector

^e^Includes religious or spiritual advisor, or social worker or counselor in any setting other than a specialty mental health setting

^f^Includes any other type of healer, such as herbalist or homeopath; participation in an internet support group; or participation in a self-help group

^g^Includes the human services sector or the CAM sector

^h^Includes any treatment listed above

*Significant difference between country income groups (HIC versus LMIC) at the 0.05 level, two-sided test

**Significant difference between severity categories at the 0.05 level, two-sided test

Treatment rates are much higher in HICs (40.5%) than LMICs (7.0%; *χ*^2^_1_ = 61.7, *p* < 0.001). Treatment setting also differs by country: OCD cases in HICs (39.0%) are much more likely than those in LMICs (3.6%) to receive treatment in a health care sector (*χ*^2^_1_ = 71.5, *p* < 0.001). Specialty mental health care is especially rare in LMICs (2.0%), even for severe cases. The treatment disparity is smaller in non-health care sectors (*χ*^2^_1_ = 4.5, *p* = 0.035) due to the fact that HIC cases are far less likely to seek treatment in these sectors (9.7%) than in health care sectors. HIC and LMIC cases do not differ in the frequency with which they receive treatment in the human services sector (4.9% vs. 1.5%, respectively; *χ*^2^_1_ = 2.2, *p* = 0.138) nor in the complementary-alternative medicine sector (7.4% vs. 2.4%, respectively; *χ*^2^_1_ = 3.6, *p* = 0.059).

### Cross-national associations of socio-demographic variables with OCD-related outcomes

Next, in a cross-national dataset aggregating across countries, we examined the relationship of each socio-demographic variable with the onset of *DSM-IV* OCD, controlling for country and for all other socio-demographic variables in the model (Table 4). Female sex (OR = 1.3) and younger age at interview (OR = 2.5–6.7) are both significantly associated with OCD onset. Education is also associated with OCD onset, with risk elevated for students (OR = 2.4) and for individuals who completed low- or high-average, rather than high, levels of education (OR = 1.8–1.9) before OCD began. Lastly, marital status predicts OCD onset, with risk elevated for individuals who were never married (OR = 1.4) before OCD began.

**Table 4:** Multivariate associations of socio-demographic predictors with *DSM-IV* OCD onset, persistence, severity, and treatment

Predictor	Lifetime onset^a^		12-month persistence^b,c^		12-month severity^b,d^		12-month treatment^b,e^	
	OR	(95% CI)	OR	(95% CI)	OR	(95% CI)	OR	(95% CI)
Gender								
Female	1.3*	(1.0–1.6)	0.9	(0.6–1.5)	1.1	(0.7–1.6)	1.3	(0.7–2.3)
Male	1.0		1.0		1.0		1.0	
*x*^2^_1_	5.3*		0.1		0.1		0.7	
Age at interview								
18–29	6.7*	(4.2–10.7)	-	-	-	-	-	-
30–44	3.7*	(2.4–5.8)	-	-	-	-	-	-
45–59	2.5*	(1.7–3.7)	-	-	-	-	-	-
60+	1.0		-		-		-	
*x*^2^_3_	73.6*		-		-		-	
Age of onset of OCD^f^	-	-	1.0	(1.0–1.0)	1.0*	(0.9–1.0)	1.0	(0.9–1.0)
*x*^2^_1_	-		0.3		4.3*		2.5	
Time since onset of OCD^f^	-	-	1.0	(1.0–1.0)	1.0	(1.0–1.0)	1.0	(1.0–1.0)
*x*^2^_1_	-		1.3		0.3		0.7	
Education								
Student	2.4*	(1.8–3.1)	1.0	(0.4–2.4)	1.1	(0.4–2.6)	0.6	(0.3–1.4)
Low	1.4	(0.8–2.3)	0.6	(0.2–2.5)	1.8	(0.5–6.3)	0.6	(0.2–1.8)
Low-average	1.9*	(1.3–2.9)	1.0	(0.3–3.1)	1.2	(0.4–3.4)	1.3	(0.5–3.7)
High-average	1.6*	(1.1–2.5)	0.7	(0.3–1.7)	3.9*	(1.4–10.3)	0.6	(0.2–1.5)
High	1.0		1.0		1.0		1.0	
*x*^2^_4_	42.6*		3.2		13.0*		4.6	
Marital status								
Never married	1.4*	(1.0–1.9)	1.1	(0.5–2.1)	1.8	(0.8–3.7)	0.4*	(0.2–0.9)
Previously married	0.6	(0.3–1.2)	2.6	(0.7–9.4)	5.5*	(1.5–19.4)	2.0	(0.5–8.4)
Currently married	1.0		1.0		1.0		1.0	
*x*^2^_2_	8.6*		2.1		7.8*		8.2*	
12-month severity								
Severe/moderate^d^	-	-	-	-	-	-	1.2	(0.6–2.2)
Mild/very mild	-	-	-	-	-	-	1.0	
*x*^2^_1_	-		-		-		0.3	
(*n*)	(21,479)		(955)		(767)		(767)	

*Abbreviations* *OCD* obsessive-compulsive disorder, *OR* odds ratio, *CI* confidence interval

^a^Based on a multivariable discrete-time survival model with person-year as the unit of analysis, estimated within the total sample of respondents who were assessed for OCD. Each OR shows the association between one predictor and the lifetime onset of OCD, controlling for country and for all other socio-demographic variables. Education and marital status were time-varying, reflecting the respondent’s status as of the year prior to OCD onset among lifetime cases

^b^Based on a multivariate person-level logistic regression model. Each OR shows the association between one predictor and a 12-month OCD-related outcome, controlling for country and for all other socio-demographic variables. Education and marital status were time-invariant, reflecting the respondent’s status at the time of OCD onset

^c^Predicted 12-month OCD within the subsample of lifetime cases whose age of onset of OCD was at least two years earlier than their age at interview

^d^Predicted a Y-BOCS score in the Severe or Moderate range (i.e., a score of at least 16 out of 40; [21]) among respondents with 12-month OCD

^e^Predicted any 12-month treatment among respondents with 12-month OCD

^f^Measured as a continuous variable

*Significant at the 0.05 level, two-sided test

None of the socio-demographic variables considered here predict the persistence of OCD. However, several variables predict greater severity of OCD, operationalized as a score in the severe or moderate (rather than mild or very mild) range on the Y-BOCS. OCD is modestly but significantly more severe for individuals with an earlier age of onset of OCD (OR = 1.0). OCD is considerably more severe for individuals who, when their OCD began, had a high-average level of education (rather than a high level; OR = 3.9) and were previously married (rather than currently married; OR = 5.5). Marital status also predicts treatment among 12-month OCD cases, with never-married individuals less likely to receive treatment than individuals who were married when OCD began.

### Cross-national associations of comorbid disorders with OCD-related outcomes

Approximately half (50.3%) of individuals with lifetime OCD also qualify for at least one of the nine other lifetime *DSM-IV* disorders assessed here (Table 5). Lifetime comorbidity is highest with mood disorders (35.0%), especially major depressive disorder (22.7%). Comorbid anxiety disorders are also common (27.1%), especially social phobia (14.6%). Conversely, only 9.0% of respondents with another lifetime mental disorder have a comorbid diagnosis of OCD. Comorbid OCD is most often reported by individuals with bipolar spectrum disorder (23.1%) and ADHD (16.6%). OCD begins first in 39.3% of comorbid cases, and this is particularly common when the comorbid condition is a substance use disorder (58.9%) or a mood disorder (58.2%). More often, OCD begins second (49.2%), and this is particularly common when the comorbid condition is ADHD (89.1%) or an anxiety disorder (50.4%). Same-year onsets occur in 11.6% of cases and are concentrated among anxiety disorders, especially panic disorder (24.0%).

**Table 5:** Conditional prevalence and temporal order of lifetime comorbid disorders with *DSM-IV* OCD

Disorder	Prevalence of lifetime comorbid disorder among respondents with lifetime OCD^a^		Prevalence of lifetime OCD among respondents with lifetime comorbid disorder^b^							
					Temporal order of OCD and comorbid disorder^c^					
					OCD first		OCD second		Same-year onset	
	%	(SE)	%	(SE)	%	(SE)	%	(SE)	%	(SE)
Any anxiety disorder	27.1	(1.8)	11.9	(0.8)	36.4	(3.7)	50.4	(3.7)	13.3	(2.6)
Panic disorder with/without agoraphobia	6.7	(1.0)	15.8	(2.2)	39.2	(8.6)	36.9	(7.8)	24.0	(8.2)
Social phobia	14.6	(1.5)	15.3	(1.4)	29.1	(5.1)	53.1	(5.0)	17.8	(4.0)
Generalized anxiety disorder	7.8	(1.1)	10.2	(1.4)	58.0	(7.4)	24.4	(5.5)	17.5	(7.1)
Posttraumatic stress disorder	9.9	(1.4)	12.0	(1.6)	52.0	(6.1)	38.2	(6.1)	9.8	(4.2)
Any mood disorder	35.0	(2.0)	12.7	(0.8)	58.2	(3.5)	30.4	(3.1)	11.4	(2.0)
Major depressive disorder	22.7	(1.7)	10.2	(0.8)	59.9	(4.7)	28.1	(3.8)	12.0	(2.4)
Bipolar spectrum disorder^d^	13.0	(1.5)	23.1	(2.3)	57.0	(5.7)	32.8	(5.4)	10.1	(3.1)
Attention-deficit/hyperactivity disorder^e^	3.1	(0.7)	16.6	(3.5)	5.7	(3.6)	89.1	(4.9)	5.2	(3.2)
Any substance use disorder	14.7	(1.4)	6.1	(0.6)	58.9	(5.6)	33.5	(5.3)	7.6	(2.7)
Alcohol abuse or dependence	12.7	(1.4)	5.9	(0.7)	60.3	(6.4)	30.3	(5.7)	9.5	(3.9)
Drug abuse or dependence	6.6	(0.9)	8.9	(1.3)	55.4	(7.4)	35.2	(6.7)	9.4	(4.6)
Any disorder	50.3	(2.3)	9.0	(0.5)	39.3	(2.6)	49.2	(2.7)	11.6	(1.7)

*Abbreviations* *OCD* obsessive-compulsive disorder, *SE* standard error

^a^Percentages reflect the proportion of respondents with the lifetime disorder in each row who also qualified for lifetime OCD

^b^Percentages reflect the proportion of respondents with lifetime OCD who also qualified for the lifetime disorder in each row

^c^Temporal order is based on the age of onset reported for OCD and for the lifetime disorder in each row

^d^Includes bipolar I disorder, bipolar II disorder, or subthreshold bipolar disorder

^e^Restricted to respondents ages 18–44 for Portugal

Compared to individuals with no prior mental disorder, individuals with any prior disorder have more than a fourfold increase in risk of subsequently developing OCD (OR = 4.4) (Additional File 1 Appendix Table 3). When tested separately in univariate models, every lifetime disorder other than alcohol use disorder significantly predicts the subsequent first onset of OCD. The odds ratios are attenuated, yet most remain significant, in a multivariate model controlling for all other comorbid disorders as well as socio-demographic variables (Table 6). OCD is most likely to develop among individuals with earlier-occurring bipolar spectrum disorder (OR = 4.4) and ADHD (OR = 3.9). Risk of OCD is also heightened among individuals with earlier-occurring social phobia (OR = 2.4), panic disorder (OR = 1.9), posttraumatic stress disorder (OR = 1.9), and major depressive disorder (OR = 1.6).

**Table 6:** Multivariate associations of temporally prior lifetime disorders with subsequent *DSM-IV* OCD onset, persistence, severity, and treatment^a^

Temporally prior disorder	Lifetime onset^b^		12-month persistence^c^		12-month severity^d^		12-month treatment^e^	
	OR	(95% CI)	OR	(95% CI)	OR	(95% CI)	OR	(95% CI)
Anxiety disorder								
Panic disorder with/without agoraphobia	1.9*	(1.0–3.5)	0.3*	(0.1–0.7)	0.6	(0.2–1.7)	3.2*	(1.1–9.9)
Social phobia	2.4*	(1.6–3.4)	1.7	(0.9–3.1)	2.1*	(1.1–4.1)	3.5*	(1.8–6.7)
Generalized anxiety disorder	1.0	(0.6–1.9)	0.7	(0.3–1.8)	4.3*	(1.4–13.2)	0.9	(0.3–2.4)
Posttraumatic stress disorder	1.9*	(1.1–3.4)	1.8	(0.8–3.9)	2.6	(1.0–6.6)	1.9	(0.7–4.7)
Mood disorder								
Major depressive disorder	1.6*	(1.1–2.4)	0.7	(0.4–1.3)	1.7	(0.9–3.4)	1.0	(0.5–2.2)
Bipolar spectrum disorder^f^	4.4*	(2.6–7.6)	1.1	(0.4–2.8)	0.8	(0.4–1.8)	1.1	(0.5–2.7)
Externalizing disorder								
Attention-deficit/hyperactivity disorder^g^	3.9*	(2.3–6.6)	0.5	(0.1–1.4)	1.3	(0.5–3.6)	1.8	(0.3–9.4)
Substance use disorder								
Alcohol abuse or dependence	1.0	(0.6–1.6)	2.9*	(1.0–8.1)	0.4	(0.2–1.1)	1.1	(0.4–3.1)
Drug abuse or dependence	1.4	(0.8–2.4)	1.5	(0.5–4.4)	0.9	(0.4–2.4)	0.8	(0.3–2.4)
(*n*)	(21,479)		(955)		(767)		(767)	

*Abbreviations* *OCD* obsessive-compulsive disorder, *OR* odds ratio, *CI* confidence interval

^a^Base models were identical to those in Table 4. Comorbid disorders were added to those models here to explore multivariate associations of temporally prior lifetime disorders with subsequent OCD onset, persistence, severity, and treatment. In the model predicting lifetime onset, comorbid disorders were time-varying, reflecting the respondent’s diagnostic status as of the year prior to OCD onset. In the models predicting 12-month prevalence, severity, and treatment, comorbid disorders were time-invariant, reflecting the respondent’s diagnostic status at the time of OCD onset. Each OR shows the association between one temporally prior disorder and an OCD-related outcome, controlling for all other disorders, socio-demographic variables, and country

^b^Predicted lifetime onset of OCD in the total sample of respondents who were assessed for OCD

^c^Predicted 12-month OCD within the subsample of lifetime cases whose age of onset of OCD was at least two years earlier than their age at interview

^d^Predicted a Y-BOCS score in the Severe or Moderate range (i.e., a score of at least 16 out of 40; [21]) among respondents with 12-month OCD

^e^Predicted any 12-month treatment among respondents with 12-month OCD, including 12-month severity as an additional covariate

^f^Includes bipolar I disorder, bipolar II disorder, or subthreshold bipolar disorder

^g^Restricted to respondents ages 18–44 for Portugal

*Significant at the 0.05 level, two-sided test

Once OCD has begun, comorbid disorders are less reliable predictors of its persistence (Additional File 1 Appendix Table 3). Compared to respondents with no comorbid disorder at the time of OCD onset, those with a comorbid disorder are no more likely to still have OCD in the 12 months before the interview (OR = 1.1). The sole exception is that persistence is heightened specifically in individuals who have an alcohol use disorder at the time of OCD onset, an effect that holds when controlling for all other disorders in a multivariate model (OR = 2.9) (Table 6). Unexpectedly, in the same multivariate model, persistence is *decreased* in individuals who have panic disorder at the time of OCD onset (OR = 0.3).

Finally, having a comorbid disorder when OCD begins predicts subsequent OCD severity (OR = 2.4) and treatment (OR = 1.9) (Additional File 1 Appendix Table 3). After controlling for all other disorders, only generalized anxiety disorder (OR = 4.3) and social phobia (OR = 2.1) are associated with increased severity of 12-month OCD (Table 6). Social phobia is also associated with treatment seeking among 12-month OCD cases (OR = 3.5), as is panic disorder (OR = 3.2).

## Discussion

The present findings are tempered by several limitations. First, we used data from representative community surveys in 10 countries across the globe, enhancing the generalizability of our results. However, as some surveys were regionally rather than nationally representative, and as prevalence estimates were highly heterogenous across these surveys and across past epidemiological studies of OCD [1], our estimates cannot be taken as fully representative of the world. There is a particular dearth of community studies of OCD in Africa that remains to be addressed. Second, to our knowledge, our large cross-national sample included more OCD cases (3665 lifetime cases, 775 12-month cases) than any previous study, increasing statistical power to detect predictors of OCD-related outcomes. That said, some countries contributed relatively few OCD cases, especially for analyses of 12-month outcomes. Third, concerns have been raised about the extent to which lay-administered structured diagnostic interviews overestimate OCD prevalence [31, 32]. It is reassuring that CIDI diagnoses of OCD are moderately to highly concordant with clinician diagnoses [6, 23, 26]. Nevertheless, many respondents who were diagnosed with 12-month OCD had very mild symptoms (i.e., Y-BOCS scores lower than 8), raising questions about their clinical significance or about possible cross-cultural differences in clinical presentation that the Y-BOCS may miss [33]. While the literature has emphasized the universality of OCD symptoms [34, 35], further work is needed to explore variability in symptom experience and expression that may have implications for diagnosis [12, 36]. Fourth, the ages at which predictors and outcomes occurred were assessed retrospectively. Although we used a probing strategy that has been shown to promote accurate dating [37] and we required time-varying predictors and outcomes to be separated by at least a year, the temporal findings should be viewed as tentative pending replication in prospective longitudinal studies. Fifth, we focused on nine common comorbid disorders that share etiologically meaningful relationships with OCD. However, other potentially comorbid disorders were omitted, including obsessive-compulsive related disorders such as hoarding disorder and body dysmorphic disorder, eating disorders, and psychotic disorders [38]. Consequently, our results likely represent lower-bound estimates of the extent of comorbidity and the ability of temporally prior mental disorders to predict OCD-related outcomes. Sixth, we assessed OCD using CIDI 3.0, which is based on *DSM-IV* diagnostic criteria. As such, we used a narrower definition of obsessions than *DSM-5*, which removed the requirements that obsessions cause marked anxiety or distress and are recognized to be a product of the individual’s own mind, and we did not perform a detailed assessment of the OCD insight specifier. Finally, we categorized Y-BOCS scores using a method designed to capture OCD cases across the full severity continuum. While this approach is appropriate for community samples, which vary widely in severity, it is less likely to be suitable for clinical samples, which typically require higher severity thresholds.

Bearing these considerations in mind, lifetime prevalence of OCD was 4.1% and 12-month prevalence was 3.0% across all surveys combined. An additional 9.5% of respondents reported lifetime experiences of obsessions or compulsions but did not qualify for an OCD diagnosis. These data are consistent with previous evidence, primarily from HICs, that OCD is more common than originally thought [1, 8] and that in the general population there is also a high prevalence of subclinical OCD [6]. The gap between early informal prevalence estimates and more recent data-based prevalence estimates may reflect that some individuals with OCD conceal their symptoms or do not view their symptoms as part of a treatable condition—factors that may in turn delay diagnosis and appropriate treatment [39]. Although OCD is often conceptualized as a discrete condition that is either present or absent, the high prevalence of subclinical presentations highlights that—as in the case of mood and anxiety disorders—there is a spectrum from wellness to illness, with even mild forms of illness potentially impairing and deserving of attention [40, 41].

The finding that OCD prevalence rates are higher in LMICs than HICs is noteworthy, given that other common mental disorders are more prevalent in HICs than LMICs [42]. Many factors could account for the variability in prevalence across countries. Despite efforts to ensure methodological consistency across surveys, variations in characteristics such as sampling frames, quality control procedures, and interview length prevented full equivalence [15]. Further, the CIDI 3.0 translation protocol, although rigorous, may not have fully overcome the complex challenges of adapting a single interview for a diverse set of languages and cultures [16]. Cultural factors may also have influenced response patterns: In LMICs, for example, stigma surrounding mental illnesses may have decreased disclosure of symptoms, while prevalent religious rituals may have increased reporting of some OCD symptoms [43]. Although higher OCD prevalence rates in lower-income countries are more likely attributable to methodological than substantive differences, OCD has been linked to infectious conditions that are more common in LMICs than HICs [44].

Notably, the proportion of cases with mild or very mild symptoms is also higher in LMICs, while the proportion of cases with moderate or severe symptoms is higher in HICs. This is consistent with the large majority of disorders assessed in the WMH surveys, for which the proportion of individuals reporting severe impairment is lower in LMICs [43] despite the relative scarcity of mental health resources and lower treatment uptake in these countries compared to HICs. It is possible that, as has been observed for other mental disorders [45], the functional impact of OCD is indeed lower in lower-income countries. This could reflect cross-national differences in role expectations (e.g., less rigid role demands) or social structures (e.g., stronger community and familial support systems). Further work is needed to probe differences in OCD prevalence and impairment across LMICs and HICs, including investigation of biopsychosocial factors that may influence differential symptom expression and reporting [46–48].

The early onset and high persistence of OCD found here are consistent with previous work emphasizing the chronic course of this condition [6]. These findings have implications for clinical services and public health policy, particularly the importance of early detection and intervention [49]. Our finding that age of onset of OCD is similar in males and females is noteworthy, given previous inconsistent findings based on smaller samples [6, 34]. However, unlike community surveys, clinical evaluation allows careful assessment of neurological features such as motor and vocal tics, and this more readily allows the delineation of an OCD subgroup that has an earlier onset, is more common in males, and involves a greater likelihood of tics [50].

Cross-nationally, fewer than one-fifth of respondents with 12-month OCD received treatment in the past year. This estimate reflects treatment for any mental health condition, suggesting that even fewer respondents received treatment specifically for OCD. In general, more severe OCD was associated with greater probability of treatment. However, treatment patterns also differed strikingly across countries. Treatment rates were much higher in HICs (40.5%) than LMICs (7.0%). Furthermore, in HICs treatment was received primarily in health care settings, most often in the specialty mental health sector, while in LMICs treatment was equally likely in health care and non-health care settings. This is concerning given that efficacious interventions for OCD, such as exposure and response prevention, are not typically available outside of specialty mental health settings. Taken together, these findings are consistent with prior descriptions of lower rates and worse quality of treatment of common mental disorders in LMICs, due to factors spanning structural barriers such as limited access to health services and attitudinal barriers such as lower perceived need for treatment [51, 52].

Multivariate models identified distinct predictors of OCD onset, persistence, severity, and treatment within the total sample. Consistent with previous work [7, 8], female sex and younger age are associated with OCD onset; so are being a student, having low-average or high-average education, and having never been married before OCD began. Extending previous work, we show that younger age of onset, high-average education, and no longer being married are associated with more severe OCD, and that never being married is associated with lower odds of treatment. That socio-demographic variables such as marital status have differing associations with different OCD outcomes makes good sense; OCD typically begins by early adulthood (so OCD onset is associated with being unmarried), OCD negatively impacts relationships (so severe OCD is associated with no longer being married), and where an individual with OCD is married a concerned spouse may insist on treatment (so lower rates of treatment are associated with never being married).

Our finding that OCD is often accompanied by comorbid mental disorders is consistent with previous clinical and epidemiological work [7, 53]. Temporal patterns may reflect typical age of disorder onset, such as the very early onset of ADHD, and may point to specific causal pathways, such as the use of alcohol to decrease anxiety symptoms. Our finding that temporally prior comorbid disorders share different associations with OCD onset, persistence, severity, and treatment is novel, underscoring the clinical importance of evaluating comorbidity, and suggesting future research directions [54]. The relationship between OCD and alcohol use, for example, is not well studied, and our finding that alcohol use disorder is positively associated with OCD persistence points to the need for clinical and research attention to this issue [55, 56]. Conversely, our finding that panic disorder predicts a less persistent course of OCD could be due to the association of panic disorder with treatment seeking [57]. Unexpectedly, temporally prior social phobia was the most consistent predictor of OCD-related outcomes. Whether social phobia is a marker for shared risk factors (e.g., behavioral inhibition; overactive performance monitoring) [58, 59], creates conditions that exacerbate OCD symptoms (e.g., social isolation) [60], or contributes directly to OCD onset, severity, and treatment seeking warrants further attention to inform the development of preventive interventions.

## Conclusions

Taken together, these data comprise the largest cross-national epidemiological study of OCD to date. Across the 10 countries included here, OCD emerges early in life, is highly persistent, and is undertreated, emphasizing the public health significance of this often-overlooked condition. At the same time, OCD is more prevalent, more persistent, less severe, and far less likely to receive treatment in LMICs than HICs, arguing for caution when generalizing research findings from HICs to LMICs. OCD has received comparatively little attention in global mental health, and further cross-national research is needed on optimal diagnostic approaches, barriers to treatment, efficacy of treatments delivered by non-specialist community health workers, and implementation of evidence-based interventions in under-resourced settings [61–64]. Research is also needed on modifiable risk factors that transcend country boundaries. Given the specificity of risk factors for OCD onset, course, severity, and treatment, each of these outcomes should be studied separately to yield more precise etiological models and intervention targets.

## Supplementary Information

Additional file 1. Appendix Table 1. World Mental Health (WMH) sample characteristics by World Bank income categories. Appendix Table 2. Prevalence of OCD Symptom Dimensions in the World Mental Health surveys. Appendix Table 3. Univariate associations of temporally prior lifetime disorders with subsequent *DSM-IV* OCD onset, persistence, severity, and treatment.

## Data Availability

Access to the cross-national World Mental Health (WMH) data is governed by the organizations funding and responsible for survey data collection in each country. These organizations made data available to the WMH consortium through restricted data sharing agreements that do not allow us to release the data to third parties. The exception is that the U.S. data are available for secondary analysis [65].

## Abbreviations

ADHD: Attention-deficit/hyperactivity disorder
CIDI 3.0: Composite International Diagnostic Interview Version 3.0
CIDI: Composite International Diagnostic Interview
CIs: Confidence intervals
DIS: Diagnostic Interview Schedule
*DSM*: Diagnostic and Statistical Manual of Mental Disorders
ECA: Epidemiological Catchment Area
HICs: High-income countries
LMICs: Low- or middle-income countries
NCS-R: National Comorbidity Survey Replication
O/C: Obsessions and compulsions
OCD: Obsessive-compulsive disorder
ORs: Odds ratios
SCID: Structured Clinical Interview for *DSM-IV*
WEIRD: Western, Educated, Industrialized, Rich, and Democratic
WMH: World Mental Health
Y-BOCS: Yale-Brown Obsessive-Compulsive Scale
