# Supplementary material for: Obsessive-compulsive disorder in the World Mental Health surveys
Source: BMC Med. 2025 Jul 9;23:416. doi: 10.1186/s12916-025-04209-5 (PMC12239380; doi:10.1186/s12916-025-04209-5)
Supplement: Supplementary file 1 — Additional file 1. Appendix Table 1. World Mental Health (WMH) sample characteristics by World Bank income categories. Appendix Table 2. Prevalence of OCD Symptom Dimensions in the World Mental Health surveys. Appendix Table 3. Univariate associations of temporally prior lifetime disorders with subsequent DSM-IV OCD onset, persistence, severity, and treatment. [file 12916_2025_4209_MOESM1_ESM.docx]

| **Appendix Table 1. World Mental Health (WMH) sample characteristics by World Bank income categories^a^** | | | | | | | | | | | | | | | | | | |
| --- | --- | --- | --- | --- | --- | --- | --- | --- | --- | --- | --- | --- | --- | --- | --- | --- | --- | --- |
|  |  |  |  |  |  |  |  |  |  | **Sample size** | | | | | | |  |  |
| **Country by income category** |  | **Survey^b^** |  | **Sample characteristics^c^** |  | **Field dates** |  | **Age range** |  | **Part 1** |  | **Part 2** |  | **Part 2 and age ≤ 44^d^** |  | **OCD sample^e^** |  | **Response rate^f^** |
| I. Low- or middle-income countries | | |  |  |  |  |  |  |  |  |  |  |  | - |  |  |  |  |
| Medellin, Colombia |  | MMHHS |  | Medellin metropolitan area. |  | 2011-12 |  | 19-65 |  | 3,261 |  | 1,673 |  | - |  | 541 |  | 97.2 |
| Iraq |  | IMHS |  | Nationally representative. |  | 2006-7 |  | 18-96 |  | 4,332 |  | 4,332 |  | - |  | 4,332 |  | 95.2 |
| Shenzhen, PRC^g^ |  | Shenzhen |  | Shenzhen metropolitan area. Included temporary  residents as well as household residents. |  | 2005-7 |  | 18-88 |  | 7,132 |  | 2,475 |  | - |  | 7,132 |  | 80.0 |
| Romania |  | RMHS |  | Nationally representative. |  | 2005-6 |  | 18-96 |  | 2,357 |  | 2,357 |  | - |  | 562 |  | 70.9 |
| Total |  |  |  |  |  |  |  |  |  | (17,082) |  | (10,837) |  | - |  | (12,567) |  | 85.9 |
| II. High-income countries | | |  |  |  |  |  |  |  |  |  |  |  |  |  |  |  |  |
| Argentina |  | AMHES |  | Eight largest urban areas of the country (approximately 50% of the total national population). |  | 2015 |  | 18-98 |  | 3,927 |  | 2,116 |  | - |  | 692 |  | 77.3 |
| Australia^g^ |  | NSMHWB |  | Nationally representative. |  | 2007 |  | 18-85 |  | 8,463 |  | 8,463 |  | - |  | 8,463 |  | 60.0 |
| Poland |  | EZOP-2 |  | Nationally representative. |  | 2018-19 |  | 18-96 |  | 11,998 |  | 4,776 |  | - |  | 1,607 |  | 63.5 |
| Portugal |  | NMHS |  | Nationally representative. |  | 2008-9 |  | 18-81 |  | 3,849 |  | 2,060 |  | 1,070 |  | 539 |  | 57.3 |
| Saudi Arabia^g^ |  | SNMHS |  | Nationally representative. |  | 2013-16 |  | 18-65 |  | 3,638 |  | 1,793 |  | - |  | 1,789 |  | 61.0 |
| Murcia, Spain |  | PEGASUS-Murcia |  | Murcia region - regionally representative. |  | 2010-12 |  | 18-96 |  | 2,621 |  | 1,459 |  | - |  | 479 |  | 67.4 |
| Total |  |  |  |  |  |  |  |  |  | (34,496) |  | (20,667) |  | (1,070) |  | (13,569) |  | 63.6 |
| III. Total |  |  |  |  |  |  |  |  |  | (51,578) |  | (31,504) |  | (1,070) |  | (26,136) |  | 70.9 |
|  |  |  |  |  |  |  |  |  |  |  |  |  |  |  |  |  |  |  |

^a^The World Bank (2012) Data. Accessed May 12, 2012 at: <http://data.worldbank.org/country>. Some of the WMH countries have moved into new income categories since the surveys were conducted. The income groupings above reflect the status of each country at the time of data collection. The current income category of each country is available at the preceding URL.

^b^MMHHS (Medellin Mental Health Household Study); IMHS (Iraq Mental Health Survey); RMHS (Romania Mental Health Survey); AMHES (Argentina Mental Health Epidemiologic Survey); NSMHWB (National Survey of Mental Health and Wellbeing); EZOP (Epidemiology of Mental Disorders and Access to Care Survey); NMHS (Portugal National Mental Health Survey); SNMHS (Saudi National Mental Health Survey); PEGASUS-Murcia (Psychiatric Enquiry to General Population in Southeast Spain-Murcia).

^c^Most WMH surveys are based on stratified multistage clustered area probability household samples in which samples of areas equivalent to counties or municipalities in the US were selected in the first stage followed by one or more subsequent stages of geographic sampling (e.g., towns within counties, blocks within towns, households within blocks) to arrive at a sample of households, in each of which a listing of household members was created and one or two people were selected from this listing to be interviewed. No substitution was allowed when the originally sampled household resident could not be interviewed. These household samples were selected from Census area data. Some WMH surveys (Poland; Murcia, Spain) used country resident or universal health-care registries to select respondents without listing households. Six of the 10 surveys are based on nationally representative household samples.

^d^The Portuguese survey used an age-restricted Part 2 sample limited to respondents ages ≤ 44.

^e^Iraq; Shenzhen, People’s Republic of China (PRC); and Australia assessed OCD in the Part 1 sample. Saudi Arabia assessed OCD in the Part 2 sample. The remaining surveys assessed OCD in a random one-third of the Part 2 sample.

^f^The response rate is calculated as the ratio of the number of households in which an interview was completed to the number of households originally sampled, excluding from the denominator households known not to be eligible either because of being vacant at the time of initial contact or because the residents were unable to speak the designated languages of the survey. The weighted average response rate is 70.9%.

^g^For purposes of cross-national comparisons we limit the sample to respondents ages > 18.

| **Appendix Table 2: Prevalence of OCD Symptom Dimensions in the World Mental Health surveys** | | | | | | | | | | | | | | | | | | | | | | | | | |
| --- | --- | --- | --- | --- | --- | --- | --- | --- | --- | --- | --- | --- | --- | --- | --- | --- | --- | --- | --- | --- | --- | --- | --- | --- | --- |
|  |  | **Prevalence of each O/C in the total sample** | | | |  | **Proportion of respondents in the row with lifetime OCD** | | | |  | **Proportion of lifetime OCD cases in the row with 12-month OCD** | | | |  | **Proportion of 12-month OCD cases in the row who are severe^a^** | | | |  | **Proportion of 12-month OCD cases in the row who are severe or moderate^b^** | | | |
| **O/C** |  | **%** | **(SE)** |  | **(n)^c^** |  | **%** | **(SE)** |  | **(n)** |  | **%** | **(SE)** |  | **(n)** |  | **%** | **(SE)** |  | **(n)** |  | **%** | **(SE)** |  | **(n)** |
| Type  Contamination |  | 4.0 | (0.2) |  | (26,136) |  | 37.8 | (2.2) |  | (1,147) |  | 72.2 | (3.6) |  | (407) |  | 4.1 | (1.8) |  | (308) |  | 38.5 | (3.8) |  | (308) |
| Harming |  | 6.5 | (0.2) |  | (26,136) |  | 38.1 | (1.9) |  | (1,755) |  | 74.3 | (2.7) |  | (627) |  | 3.4 | (1.5) |  | (470) |  | 31.0 | (3.2) |  | (470) |
| Ordering |  | 5.3 | (0.2) |  | (26,136) |  | 41.3 | (1.9) |  | (1,426) |  | 74.5 | (2.7) |  | (550) |  | 3.1 | (1.1) |  | (416) |  | 31.8 | (2.9) |  | (416) |
| Hoarding |  | 5.9 | (0.2) |  | (26,136) |  | 35.3 | (1.7) |  | (1,601) |  | 73.4 | (2.8) |  | (512) |  | 3.0 | (1.3) |  | (377) |  | 28.9 | (3.3) |  | (377) |
| Other O/C |  | 4.1 | (0.2) |  | (26,136) |  | 48.3 | (2.2) |  | (1,087) |  | 76.6 | (2.4) |  | (493) |  | 3.7 | (1.8) |  | (378) |  | 32.6 | (3.7) |  | (378) |
| Number |  |  |  |  |  |  |  |  |  |  |  |  |  |  |  |  |  |  |  |  |  |  |  |  |  |
| Exactly 1 |  | 7.1 | (0.3) |  | (26,136) |  | 16.9 | (1.2) |  | (1,898) |  | 71.0 | (3.3) |  | (316) |  | 0.4 | (0.4) |  | (228) |  | 10.9 | (2.7) |  | (228) |
| Exactly 2 |  | 3.1 | (0.1) |  | (26,136) |  | 33.5 | (2.2) |  | (822) |  | 78.1 | (3.4) |  | (248) |  | 4.1 | (2.8) |  | (193) |  | 21.1 | (4.0) |  | (193) |
| Exactly 3 |  | 1.8 | (0.1) |  | (26,136) |  | 49.2 | (3.0) |  | (483) |  | 74.1 | (4.0) |  | (221) |  | 1.9 | (1.0) |  | (159) |  | 27.4 | (4.8) |  | (159) |
| Exactly 4 |  | 1.0 | (0.1) |  | (26,136) |  | 55.3 | (4.4) |  | (285) |  | 76.0 | (5.6) |  | (146) |  | 1.1 | (0.7) |  | (117) |  | 42.8 | (6.4) |  | (117) |
| Exactly 5 |  | 0.6 | (0.1) |  | (26,136) |  | 67.4 | (5.2) |  | (177) |  | 70.8 | (7.3) |  | (106) |  | 8.8 | (5.3) |  | (78) |  | 50.6 | (8.9) |  | (78) |
| Any O/C |  | 13.6 | (0.3) |  | (26,136) |  | 30.1 | (1.0) |  | (3,665) |  | 74.1 | (1.9) |  | (1,037) |  | 2.7 | (1.0) |  | (775) |  | 25.5 | (2.2) |  | (775) |
|  |  |  |  |  |  |  |  |  |  |  |  |  |  |  |  |  |  |  |  |  |  |  |  |  |  |

Abbreviations: OCD, obsessive-compulsive disorder; O/C, obsessions/compulsions; SE, standard error.

^a^Y-BOCS score of at least 24 out of 40, which is defined as Severe in the scheme by [21].

^b^Y-BOCS score of at least 16 out of 40, which is defined as Moderate in the scheme by [21].

^c^All n’s are unweighted.

| **Appendix Table 3. Univariate associations of temporally prior lifetime disorders with subsequent *DSM-IV* OCD onset, persistence, severity, and treatment** | | | | | | | | | | | |
| --- | --- | --- | --- | --- | --- | --- | --- | --- | --- | --- | --- |
|  | **Lifetime**  **onset^a^** | |  | **12-month persistence^b,c^** | |  | **12-month severity^b,d^** | |  | **12-month treatment^b,e^** | |
| **Temporally prior disorder** | **OR** | **(95% CI)** |  | **OR** | **(95% CI)** |  | **OR** | **(95% CI)** |  | **OR** | **(95% CI)** |
| Any anxiety disorder | 4.5* | (3.5-5.8) |  | 1.1 | (0.7-1.8) |  | 3.4* | (1.9-6.0) |  | 3.0* | (1.7-5.5) |
| Panic disorder with/without agoraphobia | 4.5* | (2.8-7.3) |  | 0.5 | (0.2-1.1) |  | 1.6 | (0.6-4.4) |  | 3.7* | (1.5-9.5) |
| Social phobia | 4.3* | (3.2-5.8) |  | 1.7 | (1.0-2.9) |  | 2.1* | (1.2-3.9) |  | 3.9* | (2.1-7.3) |
| Generalized anxiety disorder | 2.1* | (1.3-3.5) |  | 0.7 | (0.2-2.5) |  | 3.4* | (1.6-7.3) |  | 1.3 | (0.5-3.4) |
| Posttraumatic stress disorder | 3.4* | (2.1-5.4) |  | 1.3 | (0.4-4.0) |  | 3.4* | (1.3-8.7) |  | 2.2 | (0.8-5.9) |
| Any mood disorder | 4.0* | (2.9-5.3) |  | 1.0 | (0.7-1.6) |  | 1.7* | (1.0-3.0) |  | 1.2 | (0.7-2.1) |
| Major depressive disorder | 2.6* | (1.8-3.7) |  | 0.9 | (0.5-1.4) |  | 2.5* | (1.3-4.6) |  | 1.1 | (0.6-2.1) |
| Bipolar spectrum disorder^f^ | 8.2* | (5.5-12.3) |  | 1.4 | (0.6-2.9) |  | 0.9 | (0.4-2.1) |  | 1.3 | (0.5-3.4) |
| Attention-deficit/hyperactivity disorder^g^ | 6.4* | (3.8-11.0) |  | 0.5 | (0.1-1.4) |  | 1.3 | (0.5-3.3) |  | 1.9 | (0.5-6.4) |
| Any substance use disorder | 1.4 | (1.0-2.1) |  | 2.4* | (1.1-5.6) |  | 0.8 | (0.3-1.9) |  | 1.2 | (0.5-3.1) |
| Alcohol abuse or dependence | 1.2 | (0.8-1.9) |  | 3.0* | (1.2-7.8) |  | 0.8 | (0.3,2.3) |  | 1.4 | (0.5-4.0) |
| Drug abuse or dependence | 2.2* | (1.3-3.6) |  | 2.1 | (0.8-5.3) |  | 1.0 | (0.3-3.2) |  | 1.1 | (0.4-3.0) |
| Any disorder | 4.4* | (3,4-5.8) |  | 1.1 | (0.7-1.6) |  | 2.4* | (1.5-3.9) |  | 1.9* | (1.1-3.4) |
| (n) | (26,136) | |  | (980) | |  | (775) | |  | (775) | |
|  |  |  |  |  |  |  |  |  |  |  |  |

Abbreviations: OCD, obsessive-compulsive disorder; OR, odds ratio; CI, confidence interval.

^a^Based on univariable discrete-time survival models with person-year as the unit of analysis, estimated within the total sample of respondents who were assessed for OCD. Each OR shows the association between one temporally prior lifetime disorder and the subsequent onset of OCD, controlling for gender, age at interview, and country.

^b^Based on univariate person-level logistic regression models. Each OR shows the association between one temporally prior lifetime disorder and a 12-month OCD-related outcome, controlling for gender, age of onset of OCD, time since onset of OCD, and country.

^c^Predicted 12-month OCD within the subsample of lifetime cases whose age of onset of OCD was at least two years earlier than their age at interview.

^d^Predicted a Y-BOCS score in the Severe or Moderate range (i.e., a score of at least 16 out of 40)^21^ among respondents with 12-month OCD.

^e^Predicted any 12-month treatment among respondents with 12-month OCD.

^f^Includes bipolar I disorder, bipolar II disorder, or subthreshold bipolar disorder.

^g^Restricted to respondents ages 18-44 for Portugal.

*Significant at the .05 level, two-sided test.
